# Assessment of Perceptions of Social Media-Based Professional Conduct Among Health Care Providers

**DOI:** 10.2196/87970

**Published:** 2026-10-23

**Authors:** Muhammad Muntazir Mehdi Khan, Edward A Joseph, Dante Gilberti, Muhammad Anees, Sricharan Chalikonda, Casey J Allen

**Affiliations:** 1 Allegheny Health Network, 320 E North Ave, Pittsburgh, PA, 15212, United States, 1 6145924651; 2 College of Medicine, Drexel University, Philadelphia, PA, United States; 3 Division of Surgical Oncology, Institute of Surgery, Allegheny Health Network, Pittsburgh, PA, United States

**Keywords:** X, Twitter, perceptions, social media, professional, health care providers, online, internet

## Abstract

An exploratory survey of health care–related X (formerly Twitter) users identified perceived challenges in digital professionalism, including exposure to misinformation, collegial disagreements, and unprofessional conduct, while highlighting gaps in institutional guidance and the need for clearer policies and digital-professionalism education.

## Introduction

Social media has reshaped how health care professionals communicate, collaborate, and engage with patients and peers [1,2]. Among these platforms, X (formerly Twitter) has become especially prominent in scientific communication and professional networking [1-5]. X has facilitated greater connectivity among scientists and physicians, enabling rapid exchange of information and online community growth through the usage of health care–related hashtags, fostering a more collaborative digital environment [1]. While such connectivity offers opportunities for education, advocacy, and patient engagement, it also brings challenges, including the spread of misinformation and concerns about professionalism [1]. In response, organizations such as the American Medical Association adopted policies that guide health care professionals’ conduct on these platforms [6,7]. Despite these efforts, unprofessional behavior persists as a key challenge. To bridge this gap, we developed a novel survey aimed at evaluating the perceptions of professionalism among health care providers on X.

## Methods

### Survey Development

A survey instrument was developed by the research team at the Allegheny Health Network (AHN), to assess perceptions of professionalism on X. Survey items were informed by recurring themes in the literature on social media professionalism and by informal input from health care professionals regarding commonly encountered forms of unprofessional activity. Before dissemination, the survey was internally reviewed by 20 participants at AHN. Participants provided structured feedback on question wording, response options, clarity, readability, interpretability, ambiguity, and relevance to the study question. This process was undertaken to refine the instrument and did not constitute formal construct validation.

The target audience of the survey were health care providers on social media platform X. The survey was designed to capture demographic characteristics and perceptions of health care providers’ engagement on X. The participation was voluntary and the data collection was anonymous. No personally identifiable information was collected and the survey platform restricted duplicate submissions during a single session. The term “unprofessional activity” was intentionally undefined and subject to respondent interpretation. The only inclusion criteria for the study were that the participants must have an X account.

### Ethical Considerations

This study was reviewed by the AHN Institutional Review Board (IRB: 2024‐274-AHNCI-AGH) and determined to meet the criteria for exempt survey research. No names, contact information, IP addresses, medical-record information, or other directly identifying information were collected.

### Survey Dissemination

The survey was disseminated via social media platform “X” using the official account of the AHN Surgical Institute between March 1, 2025, and August 30, 2025.

### Statistical Analysis

Categorical variables were summarized as frequencies and percentages, while continuous variables were presented as means with standard deviations. Internal consistency of the survey instrument was evaluated using the Cronbach α, and agreement among responses was assessed using the Kendall coefficient of concordance (W).

## Results

Among 80 respondents, the majority (n=55, 73.3%) were based in the United States. Most respondents (n=52, 65.0%) were aged 25‐34 years and 58.8% (n=47) were male. Respondents included medical students (n=34, 42.5%), researchers (n=16, 20.0%), trainees (n=14, 17.5%), and patient advocates (n=3, 3.8%).

Approximately 65.0% (n=52) of the respondents had<100 followers, majority (n=44, 55.7%) used their professional accounts daily, while 75.0% (n=60) rarely posted. Respondents primary used X for research (n=40, 50.0%), personal interests (n=40, 50.0%), and networking (n=37, 46.3%). Overall, 42.5% (n=34) of respondents reported occasional unprofessional activity and 21.3% (n=17) encountered such activity frequently. Furthermore, 76.3% (n=61) reported witnessing false or misleading information, 20.0% (n=16) encountered the sharing of sensitive medical information, and 78.8% (n=63) observed collegial disagreements (α=0.69 W=0.243) (Figure 1).

Nearly half of the respondents (n=33, 44.0%) reported a lack of institutional guidelines regarding professional social media use.

**Figure 1.**
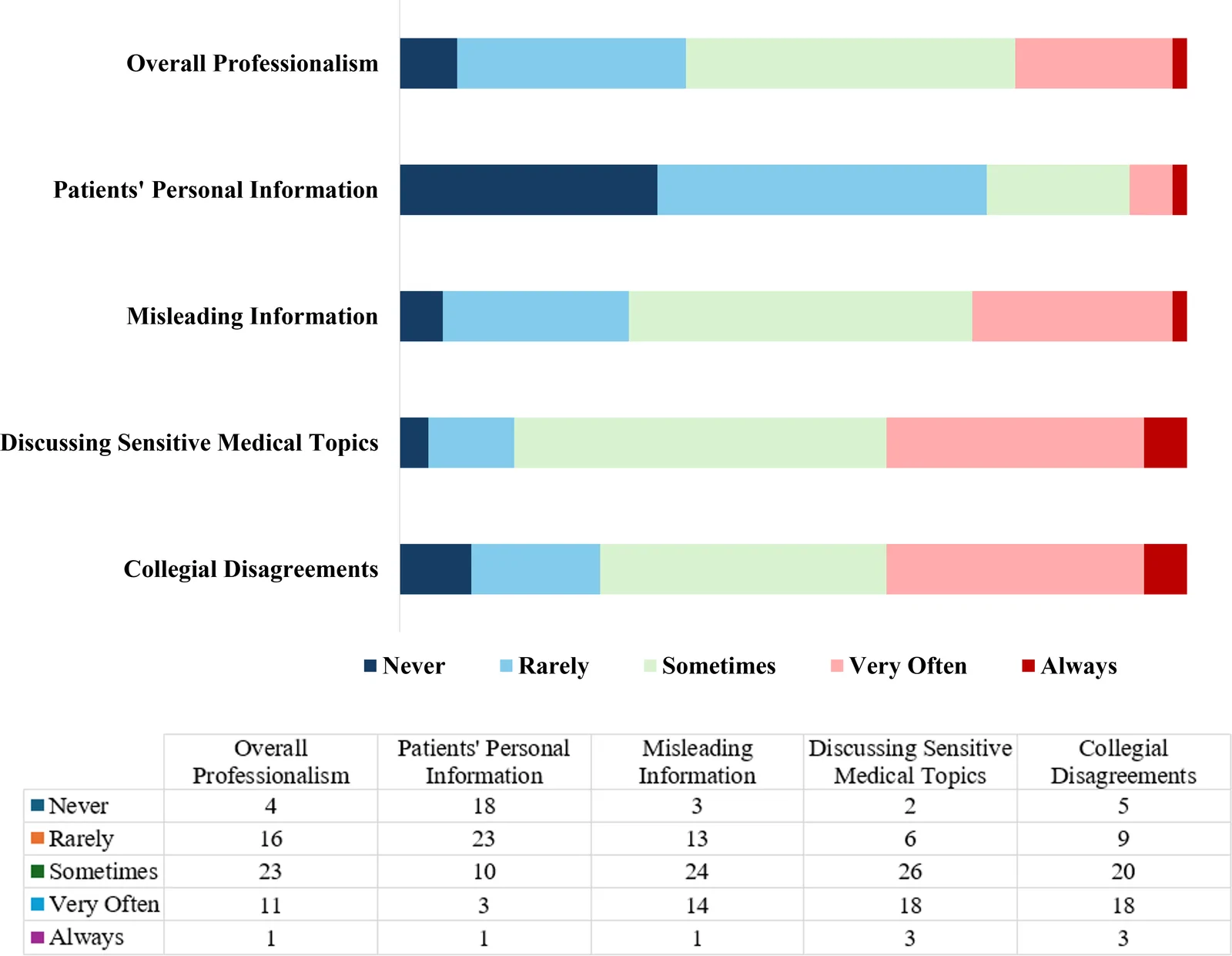
Frequency of unprofessional activity among health care providers on X/Twitter.

## Discussion

Social media platforms like X offer considerable benefits, such as facilitating rapid information dissemination, professional networking, patient engagement, and broader health care awareness [1,8]. These benefits coexist with potential risks, including misinformation, breaches of patient confidentiality, and contentious professional exchanges [9]. In our sample, 76.3% of respondents reported witnessing false or misleading information. This observation does not establish the prevalence of misinformation on X, but it suggests that respondents commonly perceived it in their online experience [9]. Likewise, the frequent observation of collegial disagreements (78.8%) illustrates that professional dialogue, while valuable for the effective exchange of thoughts and ideas, carries the risk of unprofessional discourse that may undermine the credibility of health care professionals. Explicit guidelines are essential to help health care professionals navigate the blurred boundary between personal and professional identity online [6,7]. The reported lack of such guidance reported in our study suggests that clearer, practical policies that promote digital professionalism may be useful for health care providers on social media.

This study has several limitations. Our study reliance on a single institutional X account for survey dissemination may introduce selection bias and limit generalizability. The small sample size and respondent composition limit the external validity of the findings. Response bias is also possible, as individuals with stronger opinions or more frequent exposure to social media content may have been more likely to participate. Additionally, while we captured general patterns of platform use, more granular measures of engagement, such as number of interactions or time spent on the platform were not assessed. The results showed moderate or borderline internal consistency and limited agreement among responses, likely indicating the multidimensional and subjective nature of online professionalism as the survey assessed several related but distinct domains. These metrics are interpreted in line with the hypothesis-generating nature of the instrument rather than formal validation. Furthermore, the use of subjective frequency categories without standardized definitions may have introduced variability in how respondents interpreted and selected responses. The survey did not go through a comprehensive validation process, and a formal factor analysis was not conducted due to the small sample size and exploratory nature of the study. An additional limitation of this study is the absence of predefined categories for “unprofessional activity” or “collegial disagreements.”

While social media provides significant opportunities for health care communication, our results demonstrate persistent challenges regarding unprofessional conduct and a lack of institutional guidelines. Education on best practices, integration of social media professionalism into medical training, and the establishment of clear institutional policies in conjunction with platform-specific regulations may help promote professionalism among health care providers on social media.

## Abbreviations

AHN: Allegheny Health Network
